# A novel SOX6 + melanoma cell subtype promotes early microsatellite invasion in Asian acral melanoma through fatty acid transport disorder

**DOI:** 10.1186/s13046-025-03516-2

**Published:** 2025-08-27

**Authors:** Chuan Lv, Kexin Chen, Tengjiao Wang, Junfeng Jiang, Guanghui Hu, Jianan Gu, Tao Liu, Sheng Wang, Haiying Dai, Yue Wang

**Affiliations:** 1 Department of Plastic and Reconstructive Surgery, School of Medicine, Shanghai Fourth People’s Hospital, Tongji University, Shanghai, 200434 China; 2 Department of Histology and Embryology, College of Basic Medicine, Naval Medical University, Shanghai, 200433 China; 3 Stem Cell and Regeneration Medicine Institute, Research Center of Translational Medicine, Naval Medical University, Shanghai, 200433 China; 4 Shanghai Institute of Stem Cell Research and Clinical Translation, Shanghai, 200120 China; 5 Shanghai Key Laboratory of Cell Engineering, Shanghai, 200433 China; 6 Department of Bioinformatics, Center for Translational Medicine, Naval Medical University, Shanghai, 200433 China; 7 Shanghai Key Laboratory of Signaling and Disease Research, Frontier Science Center for Stem Cell Research, School of Life Sciences and Technology, Tongji University, Shanghai, 200092 China; 8 Department of Plastic Surgery, Changhai Hospital, Naval Military Medical University, Shanghai, China; 9 School of Gongli Hospital Medical Technology, University of Shanghai for Science and Technology, Shanghai, 200093 China

**Keywords:** Acral melanoma (AM), Tumor invasion, Early microsatellites, Fatty acid transport, SOX6

## Abstract

Acral melanoma (AM) is the predominant subtype of melanoma in Asians. Early detection and prevention can significantly improve patient outcomes; however, there is a lack of effective early biomarkers for predicting AM metastasis. Here, we employed single-cell and spatial transcriptomics analyses to investigate early microsatellite lesions of AM and identify biomarkers of invasiveness in these lesions. Our results characterize a highly immunosuppressive microenvironment and metabolic process shifts in early AM microsatellite lesions that promote the metastatic potential. The transcription factor SOX6 is overexpressed in microsatellite lesions and marks a population of highly invasive melanoma cells. The pro-invasive role of overexpressed SOX6 was validated in vivo and in vitro, including its ability to enhance tumor invasion by upregulating cellular glycolysis, disrupt fatty acid transport, and increase intracellular phosphatidylcholine content. This study suggests that SOX6-overexpressing melanoma cells are the main driver subpopulation promoting early invasion of AM and establishes SOX6 and fatty acid transport processes as biomarkers and potential therapeutic targets for early melanoma metastasis.

**Supplementary Information:**

The online version contains supplementary material available at 10.1186/s13046-025-03516-2.

## Introduction

Acral melanoma (AM) is the deadliest of the cutaneous malignancies with poor prognosis upon metastasis [1, 2]. It is primarily derived from melanoma cells in sun-shielded palms, soles, and nails [3, 4]. Clinically, AM often presents at advanced stages, frequently metastasizes, and exhibits a poor prognosis. Currently, the treatment modalities for AM are similar to those of cutaneous melanoma, but AM has a lower response rate to immune checkpoint blockade (ICB) therapy, ranging from 15–20% [5–7]. Therefore, early diagnosis and prevention of AM are crucial for improving patient outcomes. Single-cell and spatial multi-omics have revealed substantial heterogeneity in the melanoma transcriptomic landscape and intricate tumor cell–cell communication [1, 3–5, 8–10]. However, these studies have mainly focused on primary or late-stage invasive AM, and the understanding of early AM remains limited.

Microsatellites were first discovered in 1981 [11] and were subsequently redefined as “discontinuous, microscopic foci of melanoma cells in the skin and/or subcutis that are separate from the primary tumor, with the intervening dermis and/or subcutis not involved by the tumor“ [12, 13]. Microsatellites have tumor metastatic potential, serve as footholds for further tumor dissemination and predict cancer recurrence with poor prognosis [14, 15]. Therefore, elucidating the mechanisms of microsatellite and corresponding biomarkers could predict the metastatic potential of early AM and provide diagnostic significance for early clinical identification.

Nevertheless, the rarity and heterogeneity of melanoma microsatellites pose significant challenges in defining common features of early AM metastasis. Beyond the substantial inter- and intra-tumoral heterogeneity in AM, the immunosuppressive microenvironment and fatty acid oxidation (FAO) metabolic reprogramming further drive metastatic progression [5, 16]. This is particularly evident in late-stage invasive AM, such as lymph node (LN) metastases, which exhibit a profoundly immunosuppressive tumor microenvironment (TME) and intricate intercellular communication networks [3, 5, 8]. As is reported, melanoma microsatellites display distinct anatomical localization and region-specific histological characteristics for early AM metastasis [4, 11] the precise cellular subpopulations involved and their regulatory mechanisms remain poorly understood. Moreover, the functional characterization of microsatellite cell subtypes are still lacking, especially for different cell types in early AM metastasis and corresponding biomarkers.

Here, we performed spatial transcriptomic sequencing (ST-seq) and single-cell RNA sequencing (scRNA-seq) to systematically investigate the heterogeneity and ecosystem of early AM microsatellites. Our results indicate that SRY-Box Transcription Factor 6 (SOX6)-positive melanoma cells in early AM metastasis are strongly invasive. In vivo and in vitro validation assays confirmed that overexpression of SOX6 upregulates glycolysis and disrupts fatty acid transport, leading to fatty acid metabolic reprogramming and increased intracellular phosphatidyl choline (PC)/phosphatidylethanolamine (PE) content. These results suggest that SOX6 expression affects cell-cell communication within the immune system, promoting cellular invasiveness and leading to tumor metastasis and poor prognosis. Our findings enhance the understanding of the mechanisms driving melanoma progression and highlight the potential for SOX6 as a therapeutic target for managing early AM invasiveness.

## Materials and methods

### Collection of clinical melanoma tissue samples

Tissue samples from 10 cases of clinical acral melanoma (AM) were obtained with informed consent and approval from the Changhai Hospital Clinical Research Ethics Committee (Approval No. CHEC2023-AM01) from the Department of Plastic Surgery , Changhai Hospital, Naval Medical University; all samples were treatment-naïve surgical resections. For immunohistochemical (IHC) staining with hematoxylin and eosin (H&E), 4 tissue samples from 3 AM patients were obtained, comprising (1) a primary in situ carcinoma without metastasis, (2) a subcutaneous metastasis without microsatellite lesions, and (3) a subcutaneous metastasis with microsatellite lesions (from which two distinct metastatic foci were analyzed). The two metastatic foci (designated M_A and M_B) from the patient with microsatellite lesions underwent spatial transcriptomic sequencing (ST-seq). Additionally, the primary in situ sample (1) and the subcutaneous metastasis sample with microsatellite lesions (3) were subjected to single-cell RNA sequencing (scRNA-seq). To validate our findings, we used a validation cohort consisting of 6 microsatellite lesion samples and 1 lymph node metastasis sample, all formalin-fixed and paraffin-embedded (FFPE). These underwent antibody staining targeting genes associated with microsatellite characteristics. This validation cohort specifically included samples illustrating the spatial relationship between microsatellite lesions and primary tumors (with accompanying HMB45 staining for verification) and samples of distant lymph node metastases.

### Microsatellite identification methodology

The identification of microsatellite lesions was rigorously performed according to the AJCC 8th edition criteria: “Microsatellites are defined as any microscopic focus of metastatic tumor cells in the skin or subcutis adjacent or deep to but discontinuous from the primary tumor. Satellite metastases are classically defined as any foci of clinically evident cutaneous and/or subcutaneous metastases occurring within 2 cm of but discontinuous from the primary melanoma. In-transit metastases are classically defined as clinically evident cutaneous and/or subcutaneous metastases occurring > 2 cm from the primary melanoma in the region between the primary melanoma and the regional lymph node basin.” This diagnostic framework was strictly implemented through histopathological evaluation of H&E-stained sections, with all determinations professionally validated by board-certified hospital pathologists.

### ST-seq library preparation

Formalin-fixed, paraffin-embedded (FFPE) samples of M_A and M_B passed the RNA quality control (DV200 > 30%). Five-micron thick sections were mounted onto a Visium Gene Expression slides (10X Genomics). H&E staining was then performed using Mayer’s hematoxylin (Millipore Sigma), bluing reagent (Dako, Agilent), and alcoholic eosin (Millipore Sigma). Stained slides were scanned under a microscope, followed by decrosslinking to release RNA sequestered by formalin. The stained slides were incubated with the Human whole transcriptome probe panel and then transferred to Cytassist (10X Genomics). Probe pairs were ligated to seal the junctions between them. The samples were treated with RNase and permeabilized to release the ligation products. The poly-A portion of the products was then captured using poly(dT) capture probes precoated on Visium slides. The probes were extended to produce spatially barcoded ligated probe products and released from the slides for indexing via Sample Index PCR. Visium Spatial Gene Expression libraries consisted of Illumina paired-end sequences flanked with P5/P7. The 16-bp Spatial Barcode and 12-bp UMI were encoded in Read 1, while Read 2 S was used to sequence the ligated probe insert.

### ST-seq data analysis

After sequencing, the reads were aligned to the Visium Human Transcriptome Probe Set (v2.0), and the expression matrix was extracted using the space ranger pipeline. Seurat (v4.1.1) [17] was then used to analyze the expression matrix. For each slide, after the spots were normalized, the top 3000 variable genes were filtered and scaled for PCA analysis, and then clusters with different resolutions were identified based on nearest neighbor analysis.

### Spatial zone division

The spatial zone division process was initiated by importing graph-based clustering results into a spatial slide loupe file, which was opened using Loupe Browser (v7.0.0). Subsequently, the tissue borders were meticulously outlined manually using the Loupe Browser’s pencil tool, ensuring accuracy through visual inspection of all graph-based unsupervised clusters. This step facilitated precise segmentation and analysis of distinct spatial regions within the tissue samples.

### Cell type decomposition

Cell type decomposition within spatial spots was achieved through Robust Cell Type Decomposition, which was integrated in the package Spacexr (v2.2.1), leveraging annotated scRNA-seq data as a reference. This process allowed the identification of cell types present in each spot along with their relative proportions, thereby providing a detailed cellular composition map across the spatial landscape.

### Stem score analysis

A trained stemness index model was built on pluripotent stem cell samples (ESC and iPSC) from the PCBC dataset using the one-class logistic regression (OCLR) machine-learning algorithm, as described in the online workflow of PanCanStem (https://bioinformaticsfmrp.github.io/PanCanStem_Web/). The corresponding mRNA expression-based stem index was used to score the spatial spot stemness levels.

### ScRNA-seq sample preparation

Tissues were transported in sterile culture dishes with 10 ml 1x Dulbecco’s Phosphate-Buffered Saline (DPBS; Thermo Fisher, Cat. no. 14190144) on ice. Dissociation enzyme [0.25% Trypsin (Thermo Fisher, Cat. no.25200-072) and 10 ug/mL DNase I (Sigma, Cat. no. 11284932001) dissolved in PBS] were applied with 5% Fetal Bovine Serum (FBS; Thermo Fisher, Cat. no. SV30087.02) to digest the tissues. Skin tissues were dissociated at 37 °C with a shaking speed of 50 r.p.m for about 40 min. The cell suspensions were filtered using a 40 μm nylon cell strainer, and red blood cells were removed using 1X Red Blood Cell Lysis Solution (Thermo Fisher, Cat. no. 00-4333-57). The dissociated cells were washed with 1x DPBS containing 2% FBS and stained with 0.4% Trypan blue (Thermo Fisher, Cat. no. 14190144) to evaluate the viability on the Countess^®^ II Automated Cell Counter (Thermo Fisher).

### 10X library preparation and sequencing

Beads with unique molecular identifiers (UMIs) and cell barcodes were loaded close to saturation, so that each cell was paired with a bead in a Gel Beads-in emulsion. After exposure to cell lysis buffer, polyadenylated RNA molecules were hybridized to the beads. The beads were then retrieved into a single tube for reverse transcription. Each cDNA molecule was tagged with a UMI. Briefly, after second-strand cDNA synthesis, adaptor ligation, and universal amplification, sequencing libraries were prepared using randomly interrupted whole-transcriptome amplification products. All remaining procedures, including the library construction, were performed according to the standard manufacturer’s protocol (CG000206 RevD). Sequencing libraries were quantified using a High Sensitivity DNA Chip (Agilent) on a Bioanalyzer 2100 and the Qubit High Sensitivity DNA Assay (Thermo Fisher Scientific). The libraries were sequenced on NovaSeq6000 (Illumina) using 2 × 150 chemistry.

### ScRNA-seq data processing

Reads were processed using the Cell Ranger 5.0.1 pipeline with default and recommended parameters. FASTQs generated from the Illumina sequencing output were aligned to the human genome, version GRCh38, using the STAR algorithm. Next, Gene-Barcode matrices were generated by counting UMIs and filtering non-cell associated barcodes. The Seurat (v4.1.1) R toolkit [18] was used for quality control and downstream analysis. All functions were run with default parameters, unless specified otherwise. Cells with fewer than 200 or more than 8,000 detected genes were excluded, where each gene had to have at least one UMI aligned in at least three cells. The expression of mitochondria genes was calculated using the PercentageFeatureSet function of the Seurat package. To normalize and enhance the analyses, we utilized the NormalizeData function from the Seurat package. Principal component analysis (PCA) was conducted on the filtered gene set, subsequently reducing the dimensionality of the dataset to the top 50 PCA components after data scaling. Harmony (v1.2.0) was used to adjust the PCA components. The harmonized PCA data was then visualized in a two-dimensional space using Uniform Manifold Approximation and Projection (UMAP). Graph-based clustering methods were also performed specifically utilizing the Louvain Method with a resolution parameter set to 0.8. The Wilcoxon Rank-Sum Test was used to identify differentially expressed genes (DEGs) for each cluster. Stacked barplot were also used illustrating cell type distribution.

### Copy number variation (CNV) score calculation

The CNV was calculated for every gene of every cell using the infercnv package (v1.16.0) [19] with B cells serving as the reference. In this context, a CNV value exceeding 1 signified a gain, while a value below 1 indicated a loss. The CNV score was calculated as the sum of squares of the deviations between each gene’s CNV value and 1. This scoring mechanism was employed to assess the malignancy potential of the clusters, with higher scores suggesting increased genomic instability or abnormality.

### Cell-cell communication analysis

ScRNA-seq data were used to analyze cell-cell communication via the CellChat tool (v1.6.1) [20]. Receptors and ligands that were expressed in more than 10% of cells within any given cell type were identified. The average expression levels of each ligand-receptor pair across various cell types were calculated to identify interactions that significantly differed between cell populations. Statistical significance was assessed with a *P*-value threshold set at < 0.01. This stringent cutoff helped to minimize false positives and ensure that only highly confident and biologically meaningful interactions were used to infer potential communication networks between different cell types.

### ScoreLentiviral vectors and plasmids

To generate stable SOX6_OE cell lines, 1 × 10^5^ A375, and SK-MEL-28 (ATCC^®^ HTB-72™) cells were cultured in DMEM and 1640 complete medium containing 5 µg/ml Polybrene, respectively. Both A375 and SK-MEL-28 cell lines are cutaneous melanoma models harboring the BRAF-V600E mutation. Subsequently, 200 µl/mL of viral supernatant (SOX6 expression virus pLVX-SOX6-Puromycin and control virus pLVX-Puromycin) was applied. After gentle mixing and incubation in a 37 °C incubator for 24 h, infected cells were selected using 1.5 µg/ml puromycin. The surviving cells were passaged for subsequent experiments. SOX6-knockdown (KD) SK-MEL-28 cell lines were performed. Lentiviral vectors encoding SOX6 shRNAs (GenePharma, Shanghai) were used to transfect melanoma cells by adding polybrene (8 mg/ml). Infected cells were selected with dose of puromycin antibiotic and cultured for 5 days before knockdown efficiency tests and functional assays. SOX6-knockout (KO) A375 stable cell lines were generated using the lentiviral CRISPR/Cas9 system. Two high-specificity sgRNAs (sgRNA1: 5′-TCACCACATAAGCCTGACGAAGG-3′ and sgRNA2: 5′-CACCACATAAGCCTGACGAAGGG-3′) of the human SOX6 gene were designed using the CRISPRscan algorithm (off-target score < 0.1) and cloned into the lentiCRISPR v2 vector (Addgene, USA). Viral supernatants were collected 48 h post-transfection, filtered (0.45 μm), and used to transduce A375 cells in the presence of 8 µg/mL Polybrene. Transduced cells underwent selection with 1.5 µg/mL puromycin (Sigma-Aldrich, USA) for 7 days. Surviving cells were single-cell cloned by limiting dilution in 96-well plates and expanded for validation. Genomic editing was confirmed by Sanger sequencing of PCR-amplified SOX6 target regions (primers: F-5′-TGAGTAAGGGGTTGAAATGCTTGT-3′;R-5′-GCCATCACCATAGTAACCTCTGAA-3′). The knockdown and overexpression efficiency were assessed by western blotting and RT-qPCR.

### RT-qPCR

Total RNA of SOX6_OE, SOX6 KO/KD and mock cells were extracted using TRIzol reagent, reverse transcribed into cDNA using a reverse transcription kit, and detected using a qPCR detection kit. β-actin was used as an internal reference. The primers were as follows: SOX6 forward primer 5′−GGTGTCACCTGGAGCAAAGA − 3′ and reverse primer 5′−CGTTGGGGACTTTACAGGCT − 3′; β-actin forward primer 5′−CATCCTCACCCTGAAGTACCC − 3′ and reverse primer 5′−AGCCTGGATAGCAACGTACATG − 3′. The relative expression levels were calculated using the 2 − ΔΔCt method.

### Western blotting

SOX6_OE, SOX6 KO/KD and mock cells were collected, and total protein was extracted using urea lysis buffer. The proteins were separated by SDS-PAGE and transferred to PVDF membranes. The membranes were blocked in 5% skim milk for 1 h, followed by incubation with primary antibodies against SOX6 (1:1000, Proteintech) and β-actin (1:5000, Proteintech) overnight at 4 °C. The membranes were then incubated with goat anti-mouse and goat anti-rabbit secondary antibodies for 1 h at room temperature. After adding the chemiluminescent substrate, the membranes were exposed to light, and images were captured and analyzed using a gel imaging system.

### Cell viability, invasion, and wound healing assays

For assessment of cell viability, the CCK8 assay (Seabiotech, shanghai, China) was used. Briefly, cells were seeded in 96-well plates at a density of 1000 cells per well and incubated for 1 day. Then, the cells were stained with CCK8 followed by determination of the OD 450 nm at 0, 1, 2, and 3 days. For invasion assays, Matrigel (BD Biosciences, Franklin Lakes, NJ, USA) was equally spread in a Transwell chamber and inoculated with 3*10^4^ cells in 200 µL serum-free DMEM. This was followed by the addition of 800 µL of medium containing 10% FBS to the lower chamber under conditions of 37 °C, 5% CO_2_, and 1% O_2_ (or 20% O_2_). After incubation, the cell suspension in the insert was removed, and cells on the upper side of the insert were wiped off with a cotton swab. The inserts were then washed, fixed, and stained with crystal violet. The invasive capacity was represented by the number of cells on the lower side of the insert, which was quantified using ImageJ software. For wound healing assays, 10^5^ A375 cells were seeded into the scratch chamber (70 µl cell suspension for each side of the scratch chamber), and then cultured overnight to adhere, followed by the removal of the scratch chamber. The medium was replaced with DMEM containing 5% FBS, and the cells were cultured in a hypoxia workstation (37 °C, 5% CO_2_, and 1% O_2_). Images were taken under a microscope at different time points (0 h, 12 h, and 24 h) using a camera attached to a light microscope (Olympus, Tokyo, Japan).

### Bulk RNA-seq library construction

Total RNA was isolated using a RNeasy mini kit (Qiagen, Germany). Strand-specific libraries were prepared using the TruSeq Stranded Total RNA Sample Preparation kit and used for strand-specific libraries (Illumina, USA) according to the manufacturer’s instructions. The libraries were sequenced on the Illumina NovaSeq 6000 (Illumina, USA). Library construction and sequencing were performed at Shanghai Biotechnology Corporation. For each sample, 33–95 M RNA-seq clean reads were obtained and mapped to GRCh38 using HISAT2 (hierarchical indexing for spliced alignment of transcripts) (v2.0.477). Sequencing read counts were calculated using Stringtie (v1.3.0). Then, the expression levels from different samples were normalized by the Trimmed Mean of M values method and converted to FPKM (Fragments Per Kilobase of transcript per Million mapped fragments). The edgeR (v3.42.4) package of R was used to analyze the differences between intergroup gene expression. The *P*-values were calculated, and the multiple hypothesis test was performed. Differentially expressed genes (DEGs) were defined as transcripts with fold change in expression greater than 2.0 and q-value less than 0.05.

### Enrichment analysis

Enrichment analysis was conducted for the DEGs using the clusterProfiler package (v4.8.3) and Metascape (https://metascape.org/gp/index.html). This analysis included Gene Ontology (GO), KEGG, HALLMARK, and GSEA pathway enrichment analyses, utilizing gene sets from the MsigDB database (https://www.gsea-msigdb.org/gsea/msigdb). The enrichment criteria included a q-value < 0.05. Heatmaps of specific genes were generated using the Pheatmap package of R. PCA analysis was performed using the scatterplot3d package of R. Enrichment analysis in DisGeNET was also used to predict targeted genes associated human diseases.

### Gene set activity analysis

Gene set activity analysis was performed using AUCell (v1.22.0) with the normalized expression data as input. The output was a matrix where each row corresponds to a gene set name and each column represents a cell barcode.

### TCGA analysis

To estimate the survival distribution of the SOX6 high and low expression groups in the TCGA cohort encompassing 458 (229 high + 229 low) patients, Kaplan-Meier analysis was performed using log-rank testing. Survival was calculated as time from TCGA specimen sampling to the time of death due to melanoma or the last follow-up time. SOX6 expression between normal melanoma (*n* = 1), primary melanoma (*n* = 104) and metastatic melanoma (*n* = 368) and was analyzed using the TCGA database. The survival analysis was performed using GEPIA2 (http://gepia2.cancer-pku.cn/#survival), and boxplots were generated using UALCAN(https://ualcan.path.uab.edu/).

### Fatty acid metabolomics

Cell samples were dissolved in chloroform: methanol (2:1, v/v) and analyzed by high-pressure liquid chromatography–mass spectrometry (HPLC-MS; Thermo, Ultimate 3000LC, Q Exactive) at a flow rate of 30 µL/min. The mobile phases were composed of acetonitrile-water mixture (60:40, v/v) containing 1% ammonium formate and acetonitrile-isopropanol mixture (10:90, v/v) containing 1% ammonium formate. The chromatographic column was Phenomenex Kinetex C18 (100 mm × 2.1 mm, 1.7 μm). Individual lipid molecular species were identified using the positive and negative electrospray ionization mode by tandem mass spectrometry. The m/z range of full scan was 250 to 1500 in positive mode, and 200 to 1500 in negative mode. The temperature was set at 350 °C. The m/z ratios of the lipids characteristic fragments and retention time of HPLC were used to identify the lipid species (tolerance of m/z value:5 ppm). NH_4_Ac and NaI were added to help to detect the neutral lipid class. The data from a total of 1528 lipid features in positive ionization mode and 1646 lipid features in negative ionization mode were normalized using Lipid Search software and Excel 2018 before statistical analysis.

### Lipid class proportion

The peak intensities of each molecular species within the same lipid class were added to represent the intensity of the individual lipid class. In total, 31 lipid classes in positive ionization mode and 40 lipid classes in negative ionization mode were identified. To calculate the overall lipid class proportion, the proportions from both ionization modes were added, resulting in a total of 52 distinct lipid classes.

### In vivo assays

For the metastasis model, 1 × 10^6^ Mock and SOX6_OE A375 cells were injected into the tail veins of 6-week-old nude athymic mice (*n* = 6 per group). Bioluminescence assays were performed by injecting 200 µL D-luciferin potassium salt (15 mg/mL; Yeasen) into the enterocoelia. The fluorescence intensities of lung metastases were observed using an IVIS Lumina III apparatus (PerkinElmer, Waltham, MA, USA).

Subcutaneous xenograft tumor models of Mock and SOX6_OE A375 cells were established by injecting 5 × 10^6^ cells per mouse into the armpits of 6-week-old nude athymic mice (*n* = 5 per group). The mice were sacrificed 4 weeks after injection, and the tumors were weighed and measured.

### Immunohistochemical staining

Clinical samples, as well as subcutaneous xenografts and lung tissues from nude mice, were embedded in paraffin and cut into 4 μm sections. The sections were deparaffinized in water and subjected to high-pressure antigen retrieval. They were then incubated with 3% hydrogen peroxide for 20 min and blocked with 10% normal goat serum to prevent nonspecific binding. Next, the sections were incubated with primary antibodies against SOX6 (1:300, Proteintech), Ki67 (1:200, ab16667), and ACSL3 (1:200, Proteintech) overnight at 4 °C. Subsequently, a streptavidin-peroxidase kit was used for detection, followed by DAB staining and counterstaining with hematoxylin.

### Statistical analysis

All experiments were performed independently at least three times. The data are expressed as the mean ± SD. The Independent Student t-test or one-way ANOVA were used to compare continuous variables between two or more groups. Differences were considered significant at a *P*-value of less than 0.05.

### Data availability

The raw sequence ST-seq and scRNA-seq data reported in this paper has been deposited in the National Genomics Data Center (https://ngdc.cncb.ac.cn/) under the accession number PRJCA035860. All the data are publicly accessible.

## Results

### Spatial transcriptomics identifies heterogeneity at different progression stages of melanoma

**Fig. 1:**
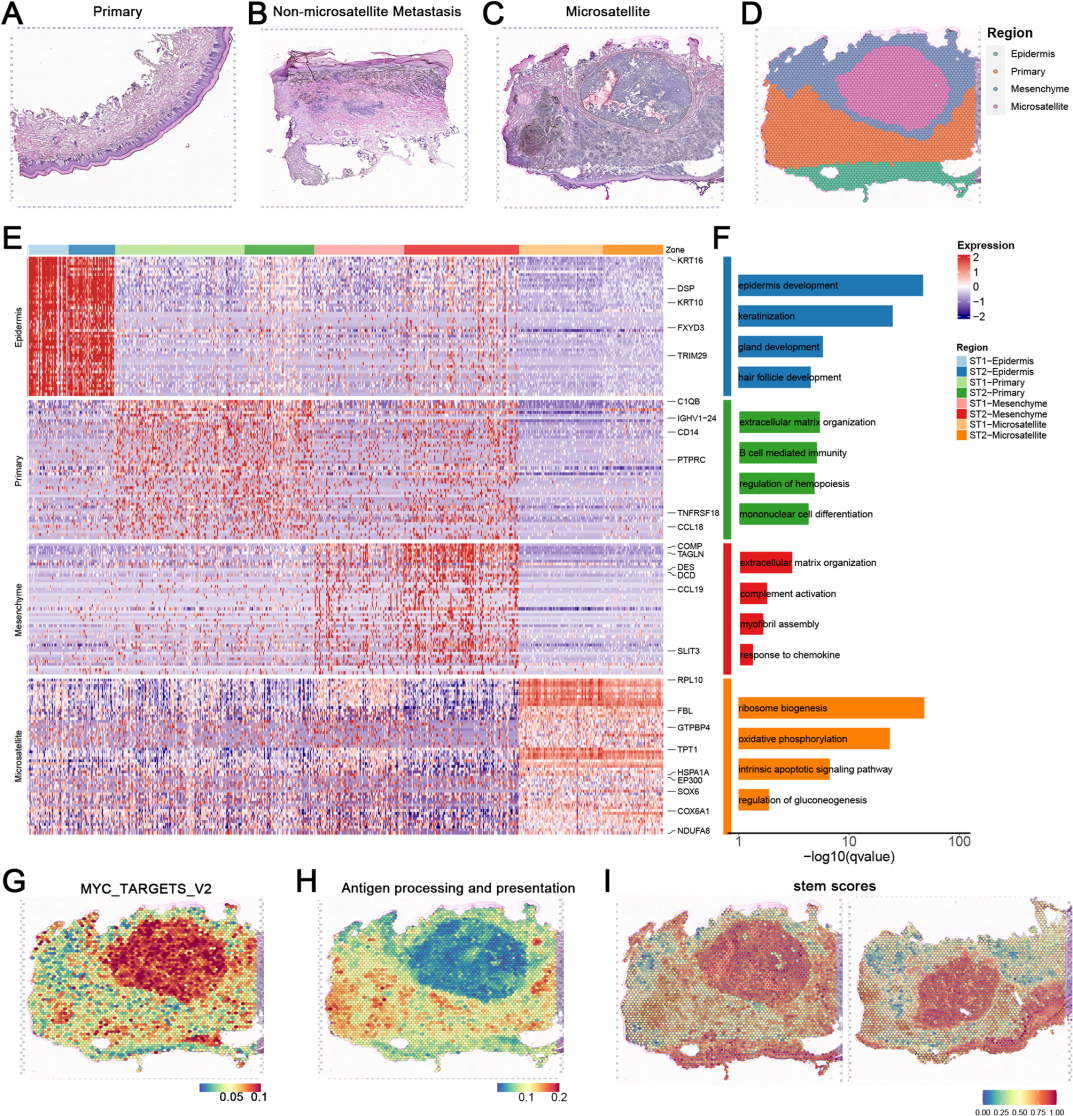
Spatial transcriptomics identifies of AM. (**A**) H&E staining of primary AM lesion sections. The primary lesion is mainly manifested as a melanoma focus confined to the epidermal layer. Scale bars = 100 μm. (**B**) H&E staining of non-microsatellites metastatic lesions AM lesion sections, which exhibited diffuse subcutaneous infiltration but lacking microsatellite formation. Scale bars = 100 μm. (**C**) H&E staining of early microsatellite lesion sections from AM (*n* = 2). Early metastatic foci formed by melanoma cells vertically invade the dermis, creating a clear histological interval between the primary and microsatellite lesions. Scale bars = 100 μm. (**D**) Spatial transcriptomics classification of an early microsatellite AM lesion section from sample M_A. The lesion was divided into four regions through marker genes, and a clear histological interval between the primary and microsatellite lesions was identified. Scale bars = 100 μm. (**E**) Display of marker genes for the four categories of regions in the spatial transcriptomics of different AM lesion sections. The main epidermal and melanoma marker genes are annotated. (**F**) GO enrichment of marker genes for the four categories of regions in the spatial transcriptomics of different AM lesion sections. Biological processes were selected based on the count of gene number (gene count > 10) and *P* value (*P* value < 1 × 10^− 2^). (**G**-**H**) Upregulation of the “MYC target gene” pathway and downregulation of the “antigen presentation” pathway are displayed using spatial transcriptomics. Immuno-suppressive features was identified in the microsatellite lesion. Scale bars = 100 μm. (**I**) The results of stemness scoring are displayed using spatial transcriptomics. A distinct tumor adaptive phenotype was identified in the microsatellite lesion. Scale bars = 100 μm

To comprehensively dissect the spatial heterogeneity of AM, we collected biopsy samples from 3 patients, utilizing spatial features to depict the tumor heterogeneity at different progression stages. Given the high invasiveness and lethality of AM, we used microsatellites to identify the dynamic evolution of early AM invasive characteristics. Here, our analysis revealed distinct metastatic phenotypes across melanoma progression stages, from primary tumors to early metastatic lesions, representing distinct progression stages: primary non-metastatic tumors (with tumor cells confined to the epidermal layer) (Fig. 1A), metastatic lesions without microsatellites (exhibiting diffuse subcutaneous infiltration but lacking microsatellite formation) (Fig. 1B), and metastatic lesions with microsatellites (showing vertical dermal invasion and distinct tumor foci) (Fig. 1C). Following the AJCC 8th edition melanoma staging criteria for microsatellite definition, our integrated analysis using H&E staining and spatial transcriptomics (ST-seq) revealed that primary lesions contained atypical melanocytes restricted to the epidermal layer (Fig. S1A; S1B; S1C), while microsatellite-positive metastases demonstrated tumor cells invading through the dermis with discontinuous tumor-stroma boundaries (Fig. 1D; S1D; S1E). In contrast, microsatellite-negative metastases showed a pattern of diffuse subcutaneous spread without focal satellite formation. This systematic approach enabled us to delineate the dynamic evolution of early invasive features in AM progression through microsatellite patterning.

For further insight, we applied ST-seq technology using the 10X Genomics Visium platform. After strict quality control, the two microsatellite slides, possessing a total of 6494 spots, were analyzed, with the median sequencing depth of a single spot of about 8920 unique molecular identifiers (UMIs) and 3236 genes. Next, we used unbiased clustering analysis to characterize the spatial diversity and classified the spots of the tumor sections based on the expression of epithelial, stromal, and melanoma marker genes. We manually checked the clusters according to the anatomical locations depicted by H&E staining and finally annotated four main regions: (1) primary tumor, (2) stroma, (3) microsatellite metastatic lesion, (4) epidermis (Fig. 1D; S1E). The H&E images and corresponding ST-seq spots mutually validated each other, confirming our spatial transcriptomics-based classification  .

Further analysis of the marker genes of each region showed that the primary lesions were enriched for pathways related to “regulation of leukocyte differentiation”, “extracellular matrix organization”, and “immunoglobulin-mediated immune response” (Fig. 1E). In contrast, the microsatellite lesions were characterized by increased expression of pathways involved in RNA synthesis and gluconeogenesis regulation processes, even mitochondrial function, indicating enhanced transcriptional activity and energy metabolism in the microsatellites, which is consistent with stronger tumor invasiveness (Fig. 1F). Additionally, the microsatellite lesions displayed the upregulation of “MYC target” pathways and downregulation of the “antigen presentation” pathway (Fig. 1G and H; S1F; S1G). In addition, the stemness score of the microsatellite lesions was higher than that of the surrounding areas, implying a more malignant and adaptive tumor subpopulation (Fig. 1I). Collectively, our findings suggest that the presence of melanoma microsatellite lesions is associated with distant invasiveness consisting with previous studies [21].

### Multifunctional melanoma cells contribute to the development of satellite lesions

While the initial spatial results revealed the localization and transcriptional differences between microsatellite foci and primary lesions, each spot in ST-seq contains a microscopic anatomical area with multiple cells. Therefore, to further characterize the microsatellite lesion-associated cell population, we carried out scRNA-seq analysis.

**Fig. 2:**
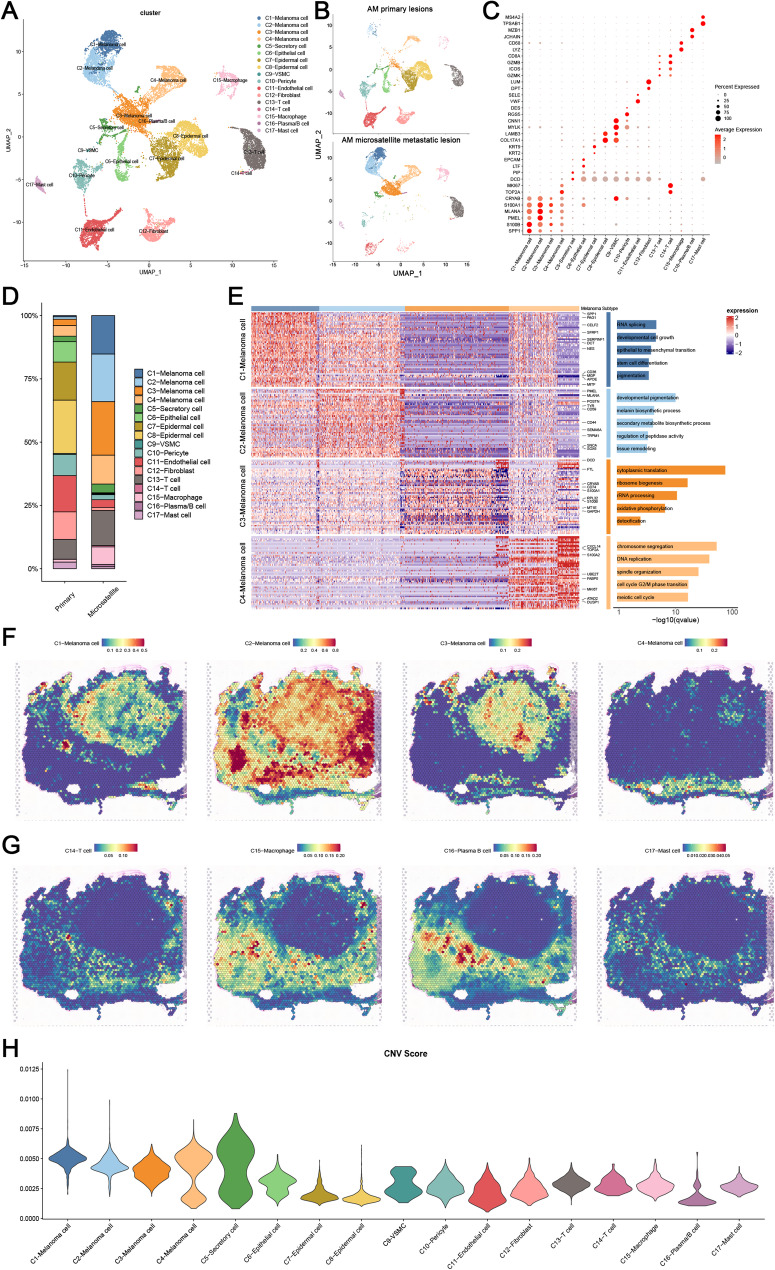
Single cell transcriptome reveled multifunctional melanoma cells in AM. (**A**) Single-cell annotation of two samples, with a total of 17 clusters annotated for 12 major cell types. (**B**) UMAP visualization showing the origin of annotated cells at different stages. (**C**) Cell type annotation using existing marker genes across the 17 major cell populations. (**D**) Stacked bar plot showing the proportion of each cell type in primary lesions and microsatellite lesions. (**E**) Display of marker genes and GO enrichment pathways for the top genes of the four melanoma cells. (**F**) Spatial locations of the four melanoma cells corresponding to gene expression profiles, demonstrating the complexity of the four cell types and the interrelationship among cells. (**G**) Spatial locations of immune cells corresponding to gene expression profiles, illustrating the distinct immune cell exhaustion within microsatellite lesions. (**H**) Violin plot displaying CNV scores across 17 major cell types, with C1 exhibiting the highest CNV burden

After standard processing and quality filtering of the original sequencing data, a total of 16,815 cells were retained for analysis. Unsupervised, graph-based clustering and UMAP visualization showed 17 different cell clusters, which were further annotated into 12 major cell types, including melanoma cells (C1-4), secretory cells (C5), epithelial cells (C6), epidermal cells (C7-8), VSMC (Vascular Smooth Muscle Cell) (C9), pericytes (C10), endothelial cells (C11), fibroblasts (C12), T cells (C13-14), macrophages (C15), plasma/B cells (C16), and mast cells (C17) (Fig. 2A-C).

Compared to primary lesions (~ 10% melanoma cells), microsatellite lesions demonstrated a significantly elevated melanoma cell proportion (> 60%, clusters C1-C4), suggesting the critical roles of melanoma cells in microsatellite pathogenesis (Fig. 2D). For additional insight into the functions of the 4 main melanoma cell populations (C1-4), we performed GO analysis for top differentially expressed genes identified in these melanoma cells (Fig. 2E). Interestingly, the 4 clusters exhibited distinct characteristics indicating complex heterogeneity within and between tumors. Cluster C1 was enriched for “developmental cell growth”, “stem cell differentiation”, and “pigmentation” pathways, primarily localized to the microsatellite lesions, suggesting a role in promoting melanoma cell maturation. Cluster C2 was enriched for “developmental pigmentation”, “melanin biosynthesis”, and “secondary metabolic processes”, potentially representing the main melanin-secreting cells. Cluster C3 was enriched in “cytoplasmic translation”, “oxidative phosphorylation”, and “detoxification” processes, which evidenced by elevated S100A1/S100B and suppressed proliferation markers, and Cluster C4 was enriched in genes related to “DNA replication” and “cell cycle processes”.

To resolve cellular composition in spatial transcriptomics, we employed the RCTD (Robust Cell Type Decomposition) algorithm to deconvolve spot-level expression data using single cell-derived reference profiles. This approach enabled quantitative mapping of cell type proportions for each visium spot. The C2 cells were significantly co-localized in both primary tumors and microsatellites. In contrast, C1 and C3 cells were mainly concentrated in microsatellites (Fig. 2F; S2A). We also mapped other cell types, such as endothelial cells, epithelial cells, and keratinocytes, to their anatomical locations (Fig. 2G; S2C). Fibroblasts were more prevalent in non-microsatellite areas, interwoven with other types of cells around the microsatellites (Fig. S2B; S2C). Consistent with H&E staining patterns, these results suggest spatial co-localization of fibroblast populations and vascular components. Importantly, compared to the immune response of the primary lesions, barely any immune infiltration was detected inside the microsatellite lesions. T cells, B cells, and macrophages could only be detected outside the microsatellite lesions, consistent with the downregulation of “antigen processing and presentation” pathway identified in the spatial transcriptomics analysis. These findings illustrated the immune cell exclusion in microsatellite metastatic lesions, which are consist with highly immunosuppressive microenvironment of LN metastasis reports relevant with invasive potential and poor prognosis [5].

To further characterize the cell subtypes, we inferred the CNV levels in each cluster, according to average expression patterns across intervals of the human genome [22, 23]. The highest level of CNV, which plays an important role in the pathogenesis and poor prognosis of tumor patients, was observed in melanoma cells, especially cluster C1^5^ (Fig. 2H; S2D). These results, together with the functional annotation results, highlight the crucial roles of the melanoma cell clusters, C1 and C2, in malignant progression.

### Expression characteristics of highly invasive melanoma subpopulations

Given that melanoma cells manifest multiple and/or overlapping/hybrid phenotypes [9] we performed subclustering analysis of the four melanoma cell clusters and examined the gene expression features (Fig. S3A). The results indicated that C1 and C2 clustered together, while C3 and C4 were separated into distinct clusters, consistent with the overarching function of C1 and C2 in melanoma maturation.

**Fig. 3:**
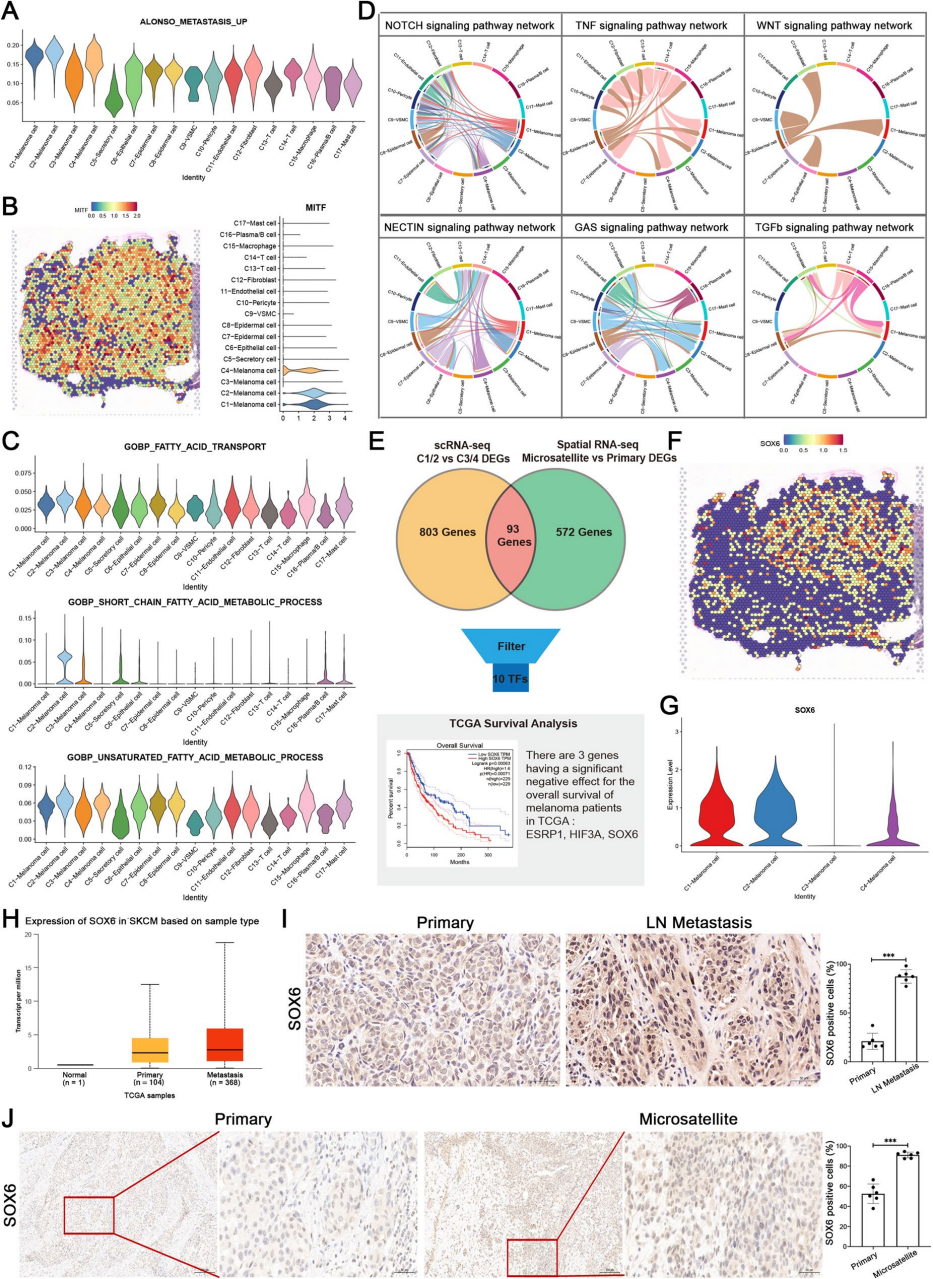
The funtional detcetion of melanoma cells. (**A**) The melanoma-specific gene signature ‘ALONSO_METASTASIS_UP’ (MSigDB) was employed to assess metastatic potential across the four cell subtypes, demonstrating elevated signature scores in C1, C2, and C4. (**B**) Spatial localization and expression profile detection of MITF in various cell types. (**C**) Pathway activity scoring (AUC Score) for “fatty acid metabolism” and “fatty acid transport”, with “short-chain fatty acid metabolism process” showing specifically high levels in C2 subpopulation. (**D**) Identification of complex intercellular communication in AM microsatellite lesions through CellChat analysis, with a focus on the “NOTCH/NECTIN”, “GAS/TNF”, and “TGFβ/WNT” pathways. (**E**) Schematic of the SOX6 screening workflow. Upregulated candidate genes were intersected between ST-seq and scRNA-seq data, followed by transcription factor (TF) screening and clinical validation using TCGA survival data. (**F**) Spatial localization of SOX6, indicating specific high expression in microsatellite lesions. (**G**) Violin plot showing SOX6 expression distribution across four melanoma cell subtypes. (**H**) Box plot showing SOX6 expression in primary and metastatic tumors in TCGA database. (**I**) Detection of SOX6 protein levels in primary and lymph node (LN) metastasis samples (*n* = 6). Scale bars = 100 μm. (**J**) Detection of SOX6 protein levels in primary and microsatellite metastasis samples (*n* = 6). Scale bars = 100 μm

Based on the invasiveness of AM, we first utilized the melanoma-specific gene signature “ALONSO_METASTASIS_UP” from the MSigDB to parse the effects of different melanoma cells on tumor metastasis (Fig. 3A) [24]. Compared to C3, C1/C2/C4 subpopulations showed higher metastasis feature, including melanoma invasion marker genes MITF and PMEL, which were highly expressed in C1/C2 (Fig. 3B; S3B). Subsequently, we conducted activity (AUC) scoring using single-cell data. Violin plots indicated that “fatty acid metabolism” and “fatty acid transport” were high in all melanoma cells. Furthermore, the “short-chain fatty acid metabolism” pathway was specifically enriched in the C2 subpopulation, suggesting an association between AM invasiveness and fatty acid metabolic dysregulation (Fig. 3C). This dysregulation likely promotes AM invasiveness through C1/C2-mediated mechanisms rather than via C3 – an metastasis-negative cluster lacking MITF expression [5].

We also used CellChat to detect intercellular communications in the microsatellite lesions (Fig. 3D). As signal senders, C1/C2 and even C4 subpopulations were observed to promote tumor-related cell type switch through the Notch/NECTIN signaling pathway, which could facilitate tumor migration. C1/C2 cells were also predicted to determine the fate of cancer-associated fibroblasts through the Notch pathway, thereby regulating the tumor microenvironment. The results are consistent with the possibility that C1/C2/C4 cells, as senders of NECTIN signals, mainly transmit signals to T cells, regulating immune processes in tumors. In addition, melanoma subpopulations were detected as receivers of immune signals such as GAS and TNF. However, the CD226 signaling pathway was involved in intercellular signal communication in the C2/C4 subpopulations in primary lesions, while no CD226-NECTIN signal interaction was detected in the melanoma subpopulations in microsatellite lesions. This could potentially explain the ineffectiveness of immune checkpoint blockade therapy in the metastatic foci of melanoma (Fig. S3D). Notably, well-known melanoma pathways, such as TGF-β and WNT, were found to mainly affect the C1 melanoma cell subtype through intercellular communication. These results indicate that epidermal tumor-promoting signals drive the transformation of C1 cells, which is consistent with the higher CNV scores observed in the C1 cluster. Integrating the localization and functional analysis of cell invasiveness of the four melanoma cells, we consider C4 to be the primary lesion in the epidermal layer. While the MITF-negative C3 cluster exhibits upregulated S100A1/S100B with downregulated proliferation markers and absence of cellular interactions, displaying a “silent” phenotype as potentially reflecting immune evasion or dormancy mechanisms [5]. With higher CNV scores and intercellular communications, C1/C2 were identified as the main driver subpopulations promoting tumor invasiveness.

To elucidate the transcriptomic features and oncogenic mechanisms of driver subpopulations promoting early metastasis, we integrated differentially expressed genes (DEGs) from microsatellite spatial transcriptomics with those from the four melanoma cell clusters (Fig. 3E). Specifically, genes upregulated in spatial transcriptomic (ST) microsatellite lesions (log2FC > 0.2; p_value_adj < 0.001) and genes upregulated in scRNA-seq C1/C2 clusters versus C3/C4 (log2FC > 0.2; p_value_adj < 0.001) were intersected. This identified 93 genes enriched in pathways including “negative regulation of immune system process”, “negative regulation of cell population proliferation”, and “developmental pigmentation” (Fig. S3E). Among these, we detected co-upregulated melanoma marker gene MLANA and metabolic genes FASN, GPX3, and APOD, suggesting metabolic reprogramming as a key factor in invasiveness. Functional annotation using DisGeNET revealed significant association of these 93 genes with metastatic melanoma (*p* = 3.16 × 10^− 5^), predicting their collective role in driving AM metastasis through differential expression in C1/C2 cells (Fig. S3F) [25].

Moreover, key transcription factors (TFs) were screened to identify the AM invasiveness driver. 10 TFs were selected form the 93 candidates, such as, HIF3A, MYC. Prognostic analysis via TCGA database highlighted three TFs with significant negative survival impact (Fig. S3G; S3H). Given the established role of SRY-box (SOX) TFs (e.g., SOX4, SOX9, SOX10) in AM metastasis [26–29] we focused on SOX6 - highly expressed in ST microsatellite lesions, elevated in C1/C2 populations, and associated with poor prognosis (*p* = 0.00063) (Fig. 3F and G; S3C). TCGA analysis even confirmed SOX6 upregulation in cutaneous melanoma (SKCM) (Fig. 3H). Distinct high-expression of SOX6 was also observed in additional biopsy samples, implicating its functional role in both AM microsatellite and even lymph node (LN) metastasis (Fig. 3I and J).

Collectively, these results demonstrate that C1/C2 melanoma cells with elevated SOX6 expression drive tumor invasiveness.

### High expression of SOX6 promotes cancer cell invasion

To verify the role of SOX6 expression in promoting tumor invasion in AM, we established SOX6-overexpressing (SOX6_OE) A375 and SK-MEL-28 stable cell line via lentivirus transduction and checked the cell proliferation and invasive capabilities. Through PCR and Western blotting assays, we confirmed that the transcription and protein levels of SOX6 in the SOX6_OE cell line were significantly up-regulated (Fig. S4A; S4B). SOX6-Knockout (SOX6_KO) A375 and SOX6-Knockdown (SOX6_KD) SK-MEL-28 stable cell lines were also used to validate the role of SOX6 in AM invasiveness. Using PCR and Western blotting assays, SOX6 were near-complete loss in SOX6_KO/SOX6_KD cells (Fig. S4C; S4D).

**Fig. 4:**
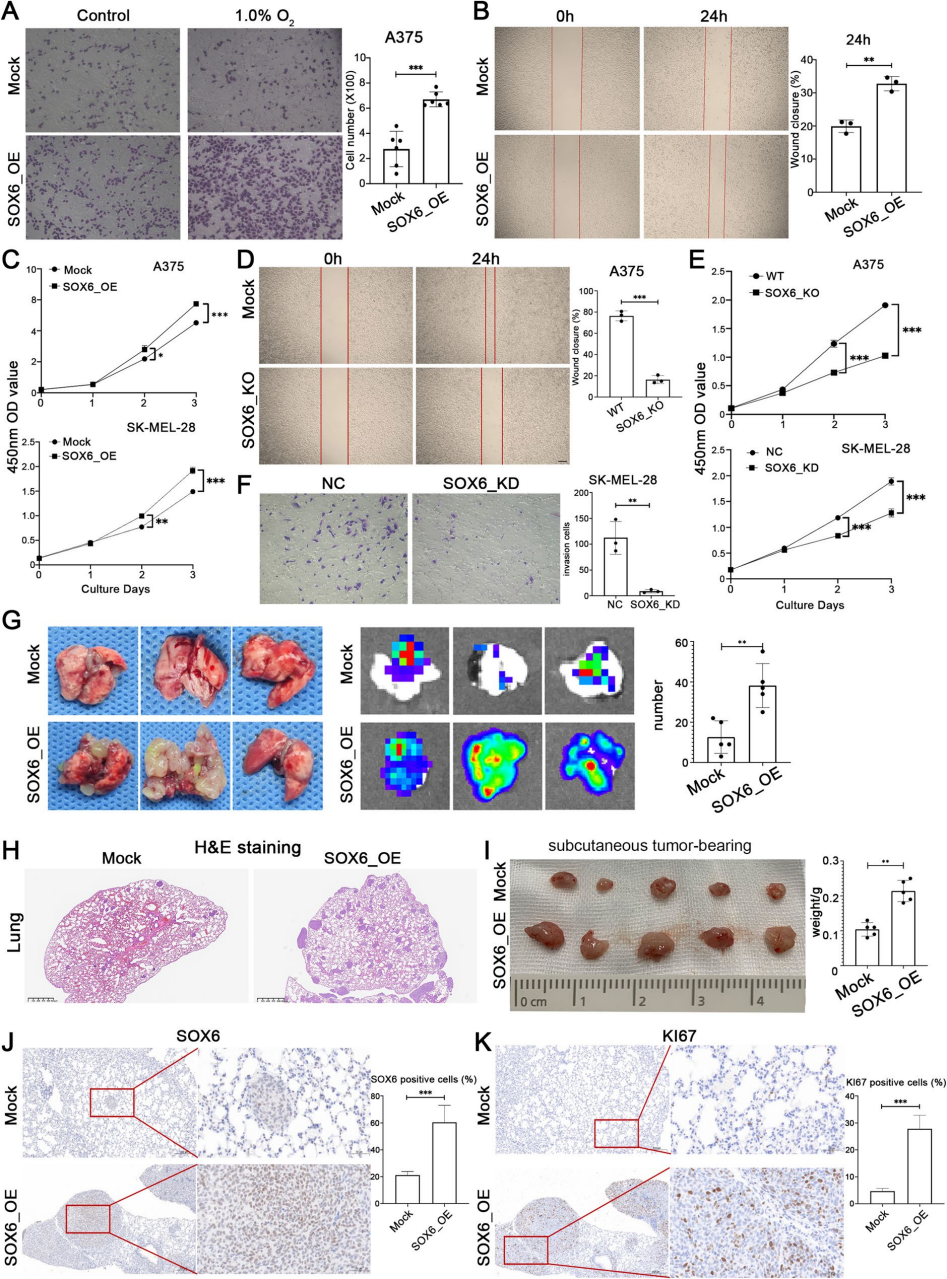
In vitro and in vivo validation of SOX_OE promoting cancer cell invasion. (**A**) Evaluation of the invasive capacity of SOX6_OE A375 cells under hypoxic conditions. (**B**) Evaluation of the migratory capacity of SOX6_OE A375 cells under hypoxic conditions. (**C**) Evaluation of the proliferative capacity of SOX6-overexpressing A375 and SK-MEL-28 cells under hypoxic conditions. (**D**) Evaluation of the migratory capacity of SOX6_KO A375 cells. (**E**) Evaluation of proliferative capacity of SOX6_KO A375 and SOX6_KD SK-MEL-28 cells. (**F**) Evaluation of the invasive capacity of SOX6_KO A375 cells. (**G**) Detection of the pro-tumor invasive ability of SOX6 overexpression in A375 cells by intravenous tail injection in mice (*n* = 5). (**H**) H&E staining of lung metastasis in mice injected with SOX6_OE A375 cells via the tail vein. Scale bars = 40 μm. (**I**) Detection of the pro-tumor occurrence and development ability of SOX6 overexpression in A375 cells by subcutaneous injection in mice (*n* = 5). (**J**) Detection of SOX6 protein levels in lung metastases of mice injected with SOX6_OE A375 cells via the tail vein. Scale bars = 100 μm. (**K**) Detection of KI67 protein levels in lung metastasis of mice injected with SOX6_OE A375 cells via the tail vein. Scale bars = 100 μm

Subsequent functional validation under physiologically relevant hypoxic conditions (present in > 90% of solid tumors) [30] revealed that SOX6 overexpression (SOX6_OE) significantly enhanced cellular invasiveness (Fig. 4A). Following 48-hour hypoxia exposure, SOX6_OE cells exhibited elevated proliferation rates and accelerated wound closure in scratch assays, indicating potentiated migratory capacity (Fig. 4B and C). Conversely, SOX6 knockout (SOX6_KO) in A375 and SOX6 knockdown (SOX6_KD) SK-MEL-28 melanoma lines demonstrated reciprocal phenotypes: markedly impaired invasiveness, diminished migration, and reduced proliferation (Fig. 4D-F). These complementary in vitro experiments collectively establish SOX6 as a critical regulator of melanoma cell proliferation, migration, and invasion.

Further in vivo validation using a tail vein injection model with SOX6_OE A375 cells in nude mice confirmed that SOX6 overexpression drives tumor progression and metastatic dissemination. After four weeks, the lungs of mice injected with SOX6_OE cells exhibited a significantly number of increased metastatic lesions [44.0 ± 9.6 vs. 12.6 ± 8.0 (tumors per lung; *p* < 0.01); (Fig. 4G)]. Histological examinations of lung tissues confirmed increased tumor size in the SOX6_OE group (Fig. 4H). Mice subcutaneously injected with the SOX6_OE also developed significantly larger subcutaneous tumors [0.10 ± 0.02 g vs. 0.02 ± 0.03 g (tumors per subcutaneous; *p* < 0.01); (Fig. 4I)]. Immunohistochemical analysis further corroborated the overexpression of SOX6 and KI67 in both pulmonary and subcutaneous SOX6_OE tumors (Fig. 4J and K; S4H). These results demonstrate that SOX6 overexpression facilitates melanoma progression and enhances metastatic potential, underscoring its role as a critical regulator in melanoma.

### High expression of SOX6 regulates metabolic reprogramming to promote tumor development

**Fig. 5:**
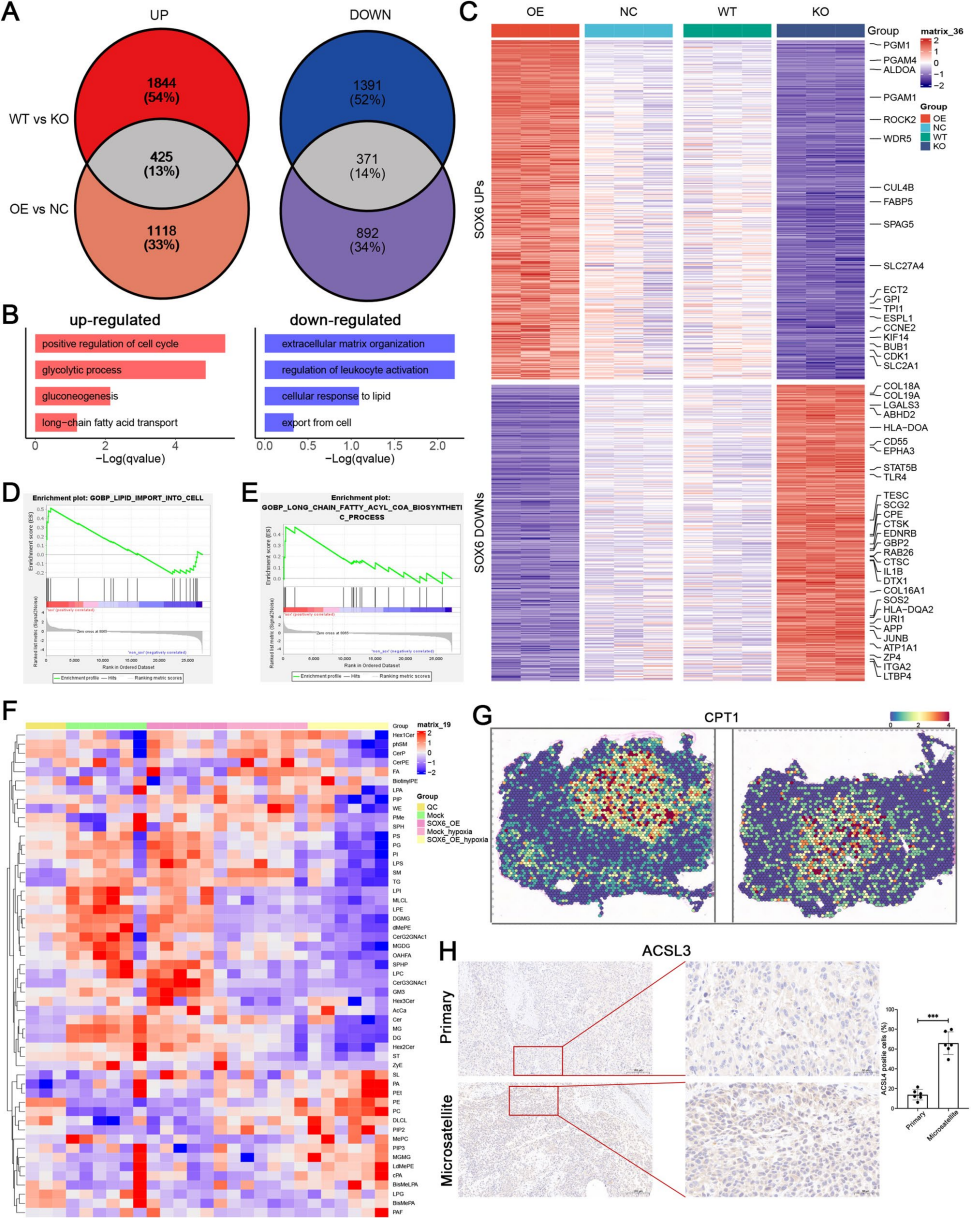
SOX6 overexpression promotes cancer cell invasion by fatty acid transport disrupt. (**A**) Transcriptome sequencing of SOX6_OE and SOX6_KO A375 cells, intersection between WT vs. KO and OE vs. NC, both up-regulated and down-regulated genes were selected for further analysis. (**B**) GO analysis of up-regulated and down-regulated genes following SOX6 overexpression. Biological processes were selected based on the count of gene number (gene count > 10) and *P* value (*P* value < 1 × 10^− 2^). (**C**) Heatmap was used for illustrated key pathways enriched in up-regulated and down-regulated genes after SOX6 dysregulation. (**D**) Specific up-regulation of “GOBP_LIPID_IMPORT_TO_CELL” process in cells overexpressing SOX6. (**E**) Specific up-regulation of long-chain fatty acid CoA biosynthesis processes in cells overexpressing SOX6. (**F**) Non-targeted fatty acid metabolomics examination of the fatty acid content in cells after SOX6 overexpression, demonstrating an increase in various fatty acids, particularly phospholipid components. (**G**) Detection of specific up-regulation of CPT1 in microsatellite lesions in ST-seq. (**H**) Detection of the key fatty acid transport gene ACSL3 in microsatellite lesions from clinical samples of different patients. Elevated ACSL3 expression were detected in microsatellite lesions (*n* = 6). Scale bars = 100 μm

To further explore the downstream mechanisms of SOX6 in tumor metastasis, we performed transcriptome sequencing to compare gene expression profiles between i) SOX6-overexpressing (OE) vs. empty vector (Mock) A375 cells; and ii) wild-type (WT) vs. SOX6-knockout (KO) A375 cells. For SOX6 up-regulated genes, which was illustrated with OE vs. NC and WT vs. KO, revealed significant enrichment in glycolipid metabolic processes and cell cycle dysregulation, such as, “Gluconeogenesis”, “glycolytis process”, “long-chain fatty acid transport” and “positive regulation of cell cycle” (Fig. 5A and B). While overlapped down-regulated pathways were enriched in skin development, including “extracellular matrix organization”, which was detected with down-regulated in both OE vs. NC and WT vs. KO. Significant “regulation of leukocyte activation” associated genes were also down-regulated consisting with immune cell exclusion in microsatellite metastatic lesions (Fig. 5C). Conversely, metabolic validation confirmed SOX6-mediated upregulation of fatty acid transporters (FABP5, SLC2A1, SLC27A6) and lipid metabolism and transport pathways, such as, “GOBP_LIPID_IMPORT_TO_CELL” and “GOMF_FATTY_ACID_ TRANSMEMBRANE_TRANSPORTER_ACTIVITY” other than “GOBP_ POSITIVE_RETGULATION_OF_FATTY_ACID_OXIDATIONL” in GSEA (Fig. 5D; S5A).

To explore the oncogenic mechanisms after SOX6 overexpression in AM microsatellite lesions, we integrated the highly expressed genes from the spatial transcriptomics and SOX6 upregulated genes. The results demonstrated significant activation of glycolytic (GPI, PGM1, PGAM1, ALDOA, HK2) and lipogenic pathways [27] with concomitant NADPH/acetyl-CoA accumulation enhancing long-chain fatty acyl-CoA biosynthesis (Fig. 5E; S5B; S5C; S5D)). These results collectively indicate that SOX6 overexpression drives glycolipid metabolic dysregulation within microsatellite microenvironments.

To validate these findings, we performed untargeted fatty acid metabolomic profiling to assess fatty acid composition following SOX6 overexpression. While hypoxia universally elevated fatty acid levels, SOX6 overexpression specifically increased the phosphatidylcholine/phosphatidylethanolamine (PC/PE) ratio while reducing lysophosphatidylcholine (LPC) content (Fig. 5F; S5F; S5G). Moreover, ACSL3 and SLC27A5 were significantly up-regulated in microsatellites, indicating fatty acid transport disorder, which induces metabolic reprogramming leading to intracellular lipid accumulation (Fig. S5H; S5I). CPT1, which was also up-regulated in microsatellites, enhances long-chain fatty acid β-oxidation and generates metabolic precursors for phosphatidylcholine (PC) biosynthesis (Fig. 5G) [31]. Moreover, this specific up-regulation of CPT1 was found to be mainly driven by C1/C2 melanoma cells in AM (Fig. S5J). PC elevation combined with LPC decrease may promote tumor invasion by activating ECM-receptor interactions and HIPPO-YAP signaling pathway, which were detected in enriched KEGG pathway of ST_high and SOX6_OE UPPER genes (Fig. S5E) [32–34]. We observed upregulation of acyl-CoA synthetase long-chain family member 3 (ACSL3), a key fatty acid transport regulator, in microsatellite lesions across multiple patient specimens (Fig. 5H). Notably, ACSL3 upregulation was also detected in lung metastases generated by tail vein injection of SOX6_OE A375 cells, as confirmed by immunohistochemistry (Fig. S5K). Collectively, our results indicate that SOX6 overexpression promotes the glycolytic process and increases the content of long-chain phospholipids such as PE/PC by disrupting fatty acid transport and fatty acid metabolism to promote tumor invasion in C1/C2 melanoma cells.

## Discussion

As early invasiveness determines the prognosis of AM [1] early diagnosis and prevention can substantially improve patient survival [35]. Here, we combined scRNA-seq with ST-seq to characterize early microsatellite lesions of AM, which are associated with metastatic potential and poor prognosis. Four melanoma cell subtypes were identified with different functions. Our results reveal that SOX6-high C1/C2 melanoma subpopulations exhibit immune cell exclusion, metabolic dysregulation and enhanced invasive capacity, similiar to advanced metastasis. Using in vivo and in vitro models, we demonstrated that high expression of SOX6 enhances cell invasiveness and promotes melanoma metastasis by reprogramming cellular metabolic processes, elevating glycolysis, dysregulating fatty acid transport, collectively reshaping the tumor microenvironment and enhancing cellular invasiveness.

Over the past decade, significant advances have been made in the field of melanoma [36, 37]. These include a new staging classification system, better understanding of the molecular biology of AM, and new immunotherapies for the treatment of advanced disease to improve patient prognosis [3, 9, 12]. Despite these advancements, our understanding of AM remains limited, underscoring the pressing demand for new therapeutic regimens that take into account its distinctive biological characteristics. The tremendous inter- and intra-tumor heterogeneity, as well as the highly immunosuppressive tumor microenvironment and complex intercellular communication networks in melanoma, have been detected in both primary and microsatellite lesions [3, 5]. Furthermore, fatty acid metabolic disorders induced by MITF have also been verified in lymph node (LN) metastasis, though these results are mainly from AM metastatic lesions [5].

Here, we utilized spatial and single cell transcriptomics, as well as anatomically defined information, to explore microsatellite foci, including the significant up-regulation of genes such as MLANA and PMEL. Similar to the spatially “cold” tumor phenotype in metastatic lesions, a high immunosuppressive microenvironment was detected in early AM microsatellites that promotes tumor metastasis [5, 10, 38]. We observed a large number of immune cells and up-regulated expression of immune response-related pathways in primary melanoma, while evident immune cell exclusion was detected in microsatellites. Using scRNA-seq, we verified that melanoma cells can manifest multiple and/or overlapping/hybrid phenotypes in early AM metastatic lesions [9]. Most melanoma cells showed higher fatty acid metabolic characteristics, especially for the C2 subtype, which was enriched in “short-chain fatty acid metabolic process” and “regulation of fatty acid transport”. C1/C2/C4 cells simultaneously exhibited stronger invasiveness and high expression of the tumor metastasis marker gene MITF, while for C3, similar to reported immunomodulatory clusters upregulated S100A1, S100B, this cell cluster promote AM metastasis by shaping a “cold” TME through microenvironmental conditioning rather than serving as metastatic diver cell clusters, highlighting the multifaceted roles of melanoma cells in AM ecosystems [5].

Combining the spatial information of cells, we found that the C2-subtype cells were widely distributed in both primary and microsatellite lesions, while C1 and C4 cells were mainly distributed in microsatellite lesions and the epidermal layer, respectively. Our results indicate that C1/C2 cells act as cellular communication media in tumors, activating NOTCH and NECTIN signals to regulate the fate determination of cells, including epithelial and fibroblast cells, which provide a mechanism for promoting carcinogenesis [39, 40]. Moreover, C1, as the only detectable receiver of WNT and TGFB signals, displays a stronger CNV malignant signal [5, 41]. These results emphasize that C1/C2 have stronger invasiveness and are likely to act as the main subpopulations driving tumor metastasis.

Notably, our analysis identified the SOX6 transcription factor, which promotes cell invasiveness, as a biomarker for early AM metastasis. Within the SOX gene family, SOX4, SOX5, SOX9 and SOX10 are established key regulators in melanoma cells establishment and function [27–29]. SOX10 loss reduces proliferation but promotes tumor invasiveness and resistance to BRAF and/or MEK inhibitors [26]. Moreover, we demonstrated that SOX6 is highly expressed in tumors such as CHOL, GBM, LGG, OV, SKCM, and STAD in the TGCA database. SOX6 were reported to play dual functional roles in oncogenesis, acting as either a tumor suppressor or oncogene depending on specific cancer types and cellular contexts [42–44]. In hematologic malignancies [42] cervical cancer [43] and multiple myeloma [45] SOX6 demonstrates tumor-suppressive properties by inhibiting proliferation, inducing cellular senescence or apoptosis, and regulating critical pathways including TGFβ2-Smad2/3 and LIN28B-MYC [43, 44]. Its downregulation in these cancers, frequently mediated through promoter hypermethylation or miRNA-dependent silencing (e.g., miR-182 in MM), contributes significantly to tumor progression and chemoresistance [45, 46]. Conversely, in Ewing sarcoma [43] prostate cancer [47] ovarian cancer [48] and endometrial cancer [49] SOX6 displays oncogenic characteristics driven by distinct mechanisms such as constitutive activation through EWSR1-FLI1 fusion protein, regulation of non-coding enhancers (SF3A1 and CCDC157), and promotion of β-catenin signaling, respectively. Through up-regulating FBXO2, SOX6 would promote ovarian cancer progression by inhibiting cell apoptosis [43, 47–49].

These opposing functional outcomes likely stem from lineage-specific co-factors, epigenetic modifications, and divergent downstream pathways [42–44, 46–49]. This context-dependent duality underscores the critical importance of considering tissue-specific regulatory mechanisms when evaluating the multifaceted roles of SOX6 in cancer biology. Though SOX6 has emerged as a promising therapeutic target across multiple cancer types, the precise mechanisms underlying its deregulation and subsequent alteration of downstream cellular events remain incompletely understood [42–49]. Particularly in acral melanoma (AM), little were known for SOX6 in promoting AM progression, compared to other SOX family members like SOX4 and SOX10 [26, 27]. Our findings demonstrate that SOX6 drives tumorigenesis and invasion through metabolic reprogramming of lipid metabolism (CPT1A, ACSL3) and glycolysis (GLUT1, LDHA), as well as immune evasion mechanisms. Notably, the C1 and C2 cell subpopulations - characterized by high SOX6 expression - were functionally validated through SOX6 overexpression (SOX6_OE) and knockout (SOX6_KO) models to promote glycolytic dysregulation in melanoma cells [27].

For additional validation, we established an in vivo xenograft model by injecting control or SOX6_OE cells into mice. Our results indicate the key coordinator role of SOX6 in promoting the invasive capacity of melanoma cells and driving metastasis. Overexpression and knockout of SOX6 dys-regulated the glycolytic process and promoted disordered fatty acid transport, resulting in the accumulation of intracellular lipid substances. SOX6 overexpression modulated the components of long-chain lipid substances, especially phospholipids such as PC/PE. Increased PC is typically resulted from decreased LPC content, leading to fatty acid transport dysfunction and has been shown to promote tumor invasiveness by activating HIPPO-YAP and EMT pathways, ultimately regulating the tumor cell cycle [31–34]. Therefore, our results are consistent with a mechanism for SOX6 in promoting melanoma via regulation of fatty acid metabolism.

Importantly, mice injected with SOX6_OE cells showed a significant increase in the pulmonary tumor burden. The SOX6_OE-driven metastatic lesions exhibited enhanced tumor heterogeneity, highlighting SOX6 function in driving melanoma cell differentiation. Furthermore, cell membrane protein-related genes such as CPT1, ACSL3, and SLC27A5 were up-regulated after SOX6 overexpression and were specifically highly expressed in microsatellites, as demonstrated by spatial transcriptomics. These fatty acid transport-related genes modulate lipid metabolism and may promote the up-regulation of MITF-mediated fatty acid oxidation [50]. Notably, these DEGs were also accompanied by up-regulation of the downstream long-chain fatty acid acetyl-CoA synthesis process to provide energy for tumor development. The up-regulation of CPT1, ACSL3, SLC27A5, and associated PC content was primarily detected in SOX6 + C1/C2 melanoma cells, suggesting that fatty acid transport, rather than fatty acid oxidation, constitutes the primary mechanism in AM metastasis. Due to the rarity of AM microsatellite lesions and the strong inter- and intra-tumor heterogeneity, further expansion of the sample cohort is warranted to more robustly elucidate the developmental and metastatic characteristics of this disease , which limits the interpretation of our study. Therefore, we employed a validation cohort, which verified the high expression of ACSL3 in the primary and microsatellite lesions of AM patients, even higher in microsatellite lesions. Together, our data offer a rationale for the clinical testing of SOX6 targeting and downstream fatty acid membrane transport processes as a novel approach to targeted therapy for AM.Crucially, matching primary tumors and microsatellite metastases from the same patients are required in future scRNA-seq and ST studies. Despite the challenges involved, this approach is essential to address the confounding effect of inter-patient heterogeneity. 

Altogether, SOX6 take part in oncogenic role in AM aligns with the established functions of other SOX family members in metastasis development programs, suggesting a conserved selection of lineage-specific mechanisms. We propose that SOX6 + tumor-promoting effects in AM originate from its ability to rewire lipid metabolism - a mechanism repurposed from its physiological functions - thereby highlighting its potential as a therapeutic target for cancer treatment in AM.

## Conclusion

In this study, we characterize the immune cell exclusion and metabolic process disorders in microsatellite lesions of AM through integrated single-cell RNA sequencing (scRNA-seq) and spatial transcriptomics (ST-seq). Our results reveal an invasive C1/C2 melanoma cells subpopulations marked by high SOX6 expression. SOX6 were demonstrated to enhance cell invasiveness by elevating glycolysis, and regulating tumor fatty acid transport to increase intracellular PC and PE content, thereby affecting the tumor microenvironment. Our findings provide mechanistic insights into AM microsatellite progression and propose SOX6-targeted inhibition—along with downstream fatty acid transport modulation—as a novel therapeutic strategy for AM.

## Supplementary Information

Below is the link to the electronic supplementary material.

Supplementary Material 1

Supplementary Material 2

Supplementary Material 3

Supplementary Material 4

Supplementary Material 5

Supplementary Material 6

Supplementary Material 7

## Data Availability

The raw sequence ST-seq and scRNA-seq data reported in this paper has been deposited in the National Genomics Data Center (https://ngdc.cncb.ac.cn/) under the accession number PRJCA035860. All the data are publicly accessible.
