# Supplementary material for: A novel SOX6 + melanoma cell subtype promotes early microsatellite invasion in Asian acral melanoma through fatty acid transport disorder
Source: J Exp Clin Cancer Res. 2025 Aug 27;44:254. doi: 10.1186/s13046-025-03516-2 (PMC12382077; doi:10.1186/s13046-025-03516-2)
Supplement: Supplementary file 1 — Supplementary Material 1 [file 13046_2025_3516_MOESM1_ESM.docx]

**Figure and figure legend**


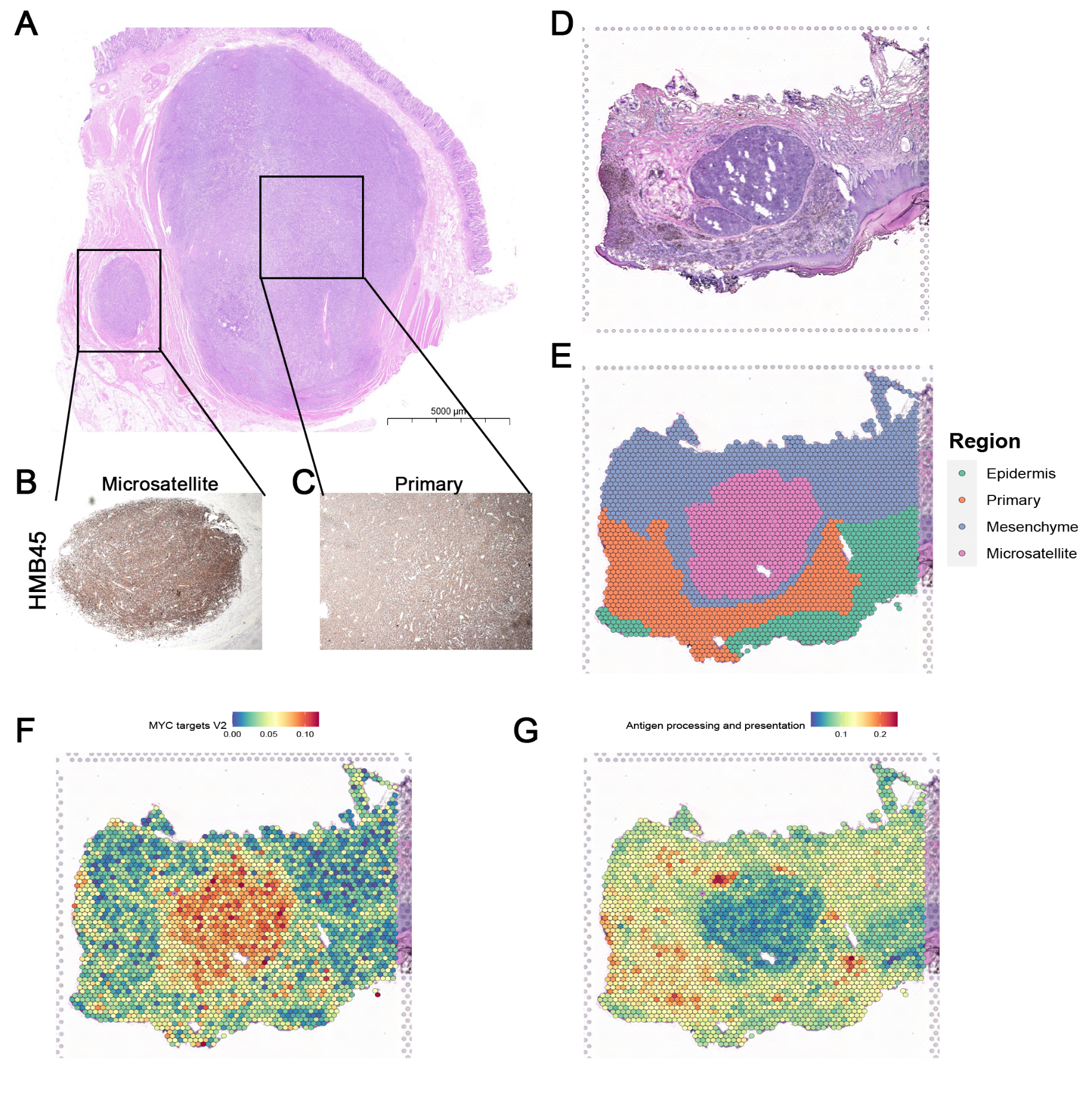


**Supplementary Figure 1.** **Spatial transcriptomics validation using different biopsy samples.**

(A) H&E staining of primary and microsatellite lesion sections from patients in the validation cohort. Scale bars = 40 μm.

(B-C) HMB45 staining of melanoma marker genes in different progression stages of AM lesion sections from patients. Scale bars = 100 μm.

(D) H&E staining of primary and microsatellite lesion sections from sample M_B. Scale bars = 100 μm.

(E) Spatial transcriptomics analysis of primary and microsatellite lesion sections from sample M_B.

(F-G) Upregulation of the "MYC target gene" pathway and downregulation of the "antigen presentation" pathway are displayed using spatial transcriptomics. Scale bars = 100 μm.


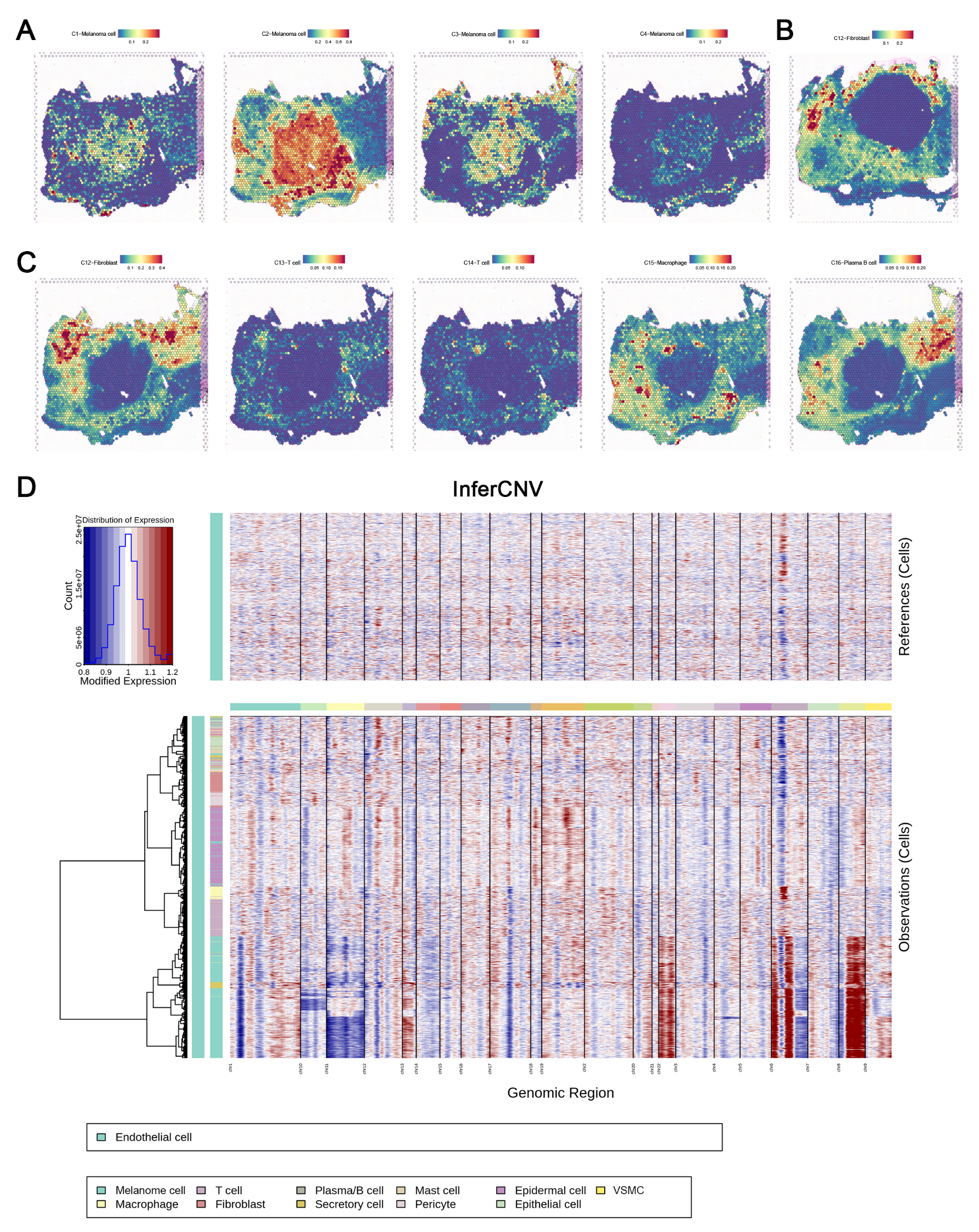


**Supplementary Figure 2. Spatial transcriptomic and CNV of multifunctional melanoma cells.**

1. Spatial locations of the four types of melanoma cells corresponding to gene expression profiles in M_B, demonstrating the complexity and interrelationship among cells.
2. Spatial locations of the fibroblasts corresponding to gene expression profiles in M_A.
3. Spatial locations of fibroblasts and immune cells corresponding to gene expression profiles in M_B, illustrating the distinct exhaustion of both fibroblasts and immune cells within the microsatellite lesions.

(D) Heatmap visualization of CNV profiles across distinct cell types.


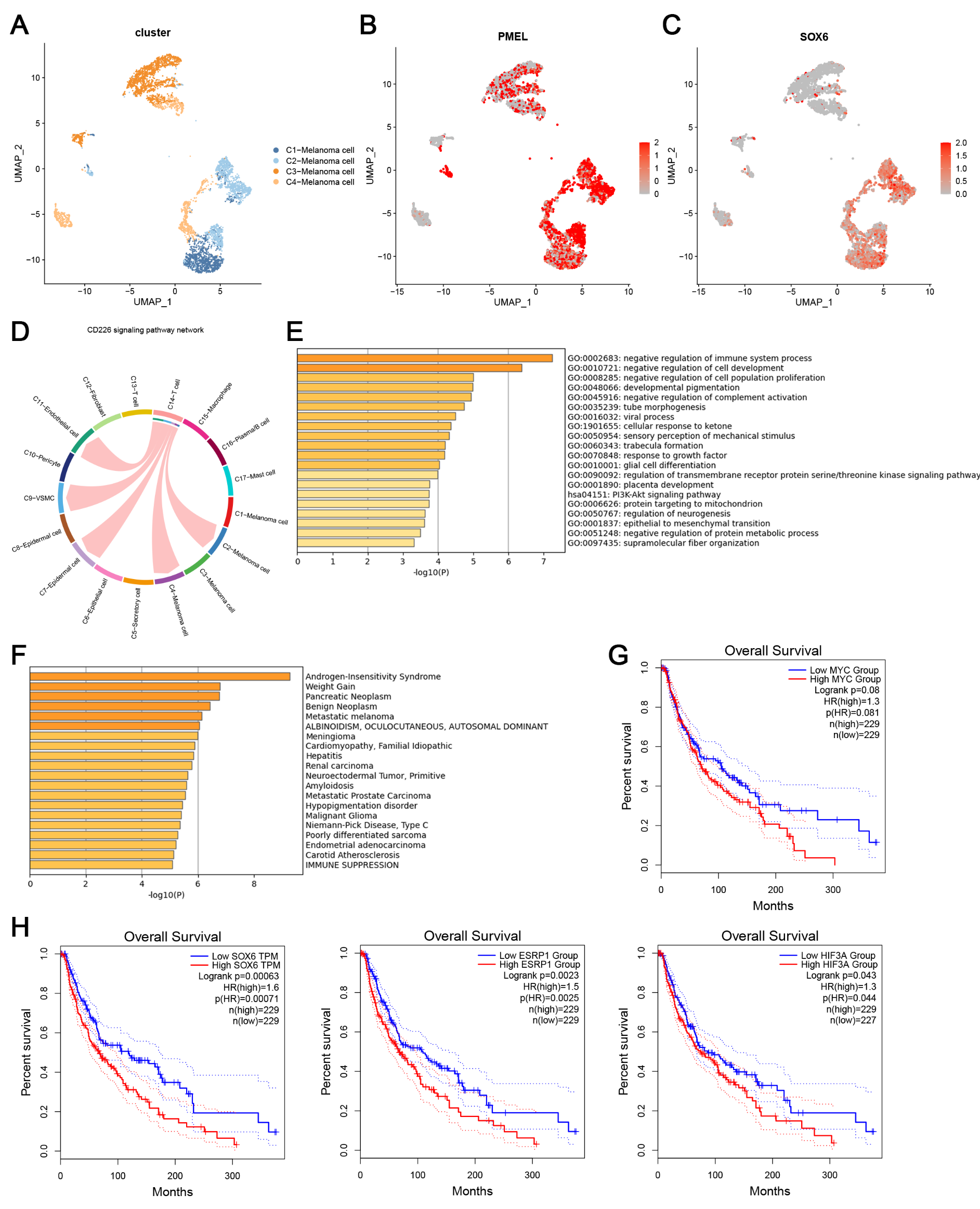


**Supplementary Figure 3. Re-clustering of the four types of melanoma cells.**

(A) Re-clustering of the four melanoma cells, with C1/C2 and a small portion of C4 clustering together.

(B) Expression of PMEL in four types of melanoma cells, mainly detectable in the C1/C2 subtypes and some C3 subtypes.

(C) Expression of SOX6 in four types of melanoma cells, primarily detectable in the C1/C2 types.

(D) CellChat results for CD226 signaling, showing detectable signal exchange processes in the C2/C4 melanoma cell subpopulations.

(E) GO enrichment of 93 candidates genes were performed using Metascape. Biological processes and KEGG were selected based on the count of gene number (gene count > 10) and P value (P value < 1 × 10^−2^).

(F) Enrichment analysis in DisGeNET was also used to predict targeted genes associated human diseases.

(G-H) Poor prognosis analysis was performed for MYC and SOX6, ESRP1, HIF3A in TCGA database using GEPIA.


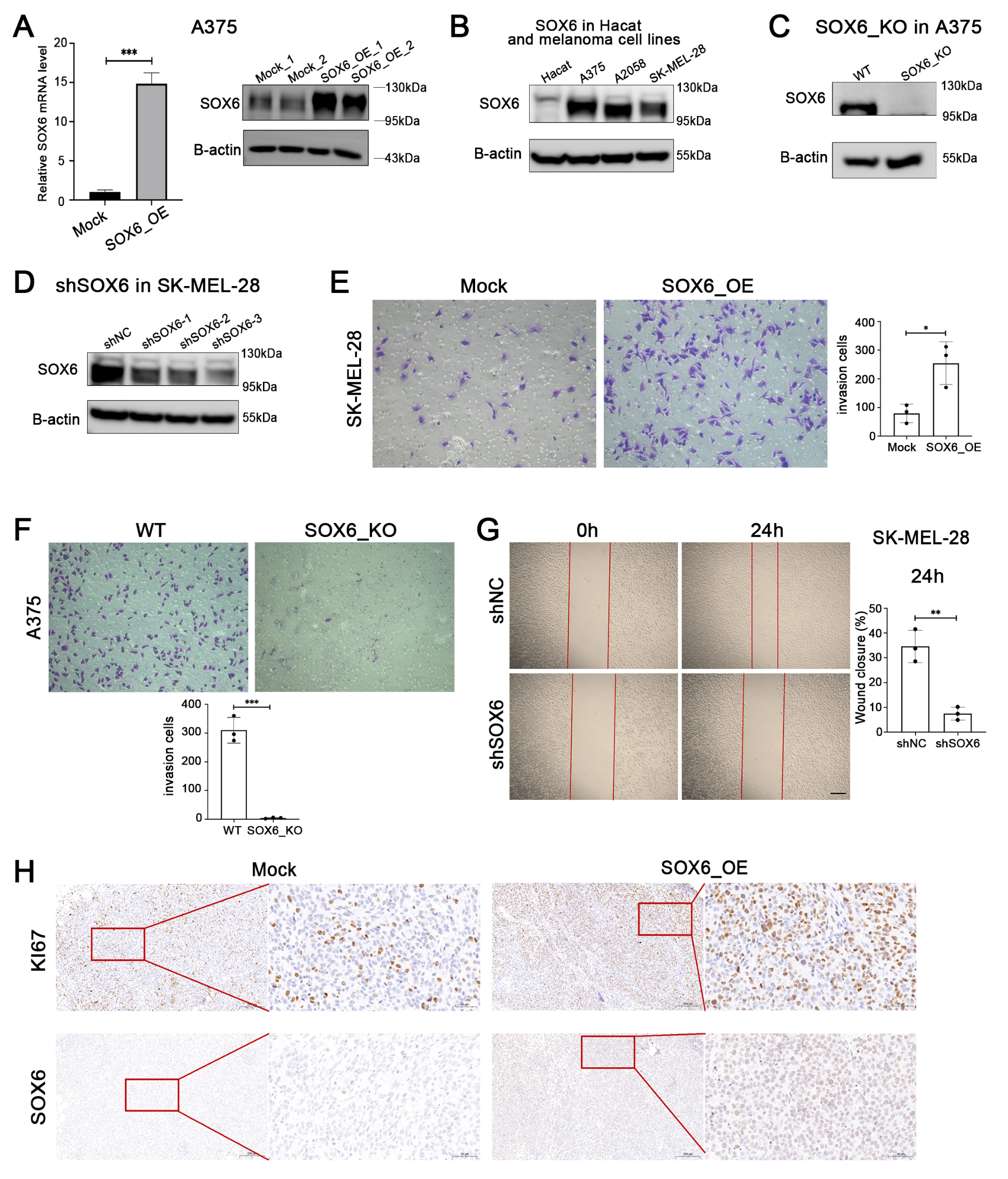


**Supplementary Figure 4. H&E staining using SOX6_OE cell line injected mice.**

1. RT-qPCR and Western blot detection of the SOX6_OE A375 cell line.
2. Western blot detection of the SOX6 levels in A375, A2058 and SK-MEL-28 cell lines.
3. Western blot detection of the SOX6 level in SOX6_KO A375 cell line.
4. Western blot detection of the SOX6 level in SOX6_KD SK-MEL-28 cell line.
5. Transwell invasion assays demonstrated that SOX6_OE SK-MEL-28 cells exhibited significantly enhanced invasive capacity compared to controls (n = 3). Scale bars = 100 μm.
6. Transwell assays revealed significantly reduced invasion capacity in SOX6_KO A375 cells compared to wild-type controls (n = 3). Scale bars = 100 μm.
7. Scratch assays demonstrated significantly impaired migration in SOX6_KD SK-MEL-28 cells (n = 3). Scale bars = 100 μm.
8. Detection of SOX6 and Ki67 protein levels in tumors of mice subcutaneously injected with SOX6_OE A375 cells. Scale bars = 100 μm.


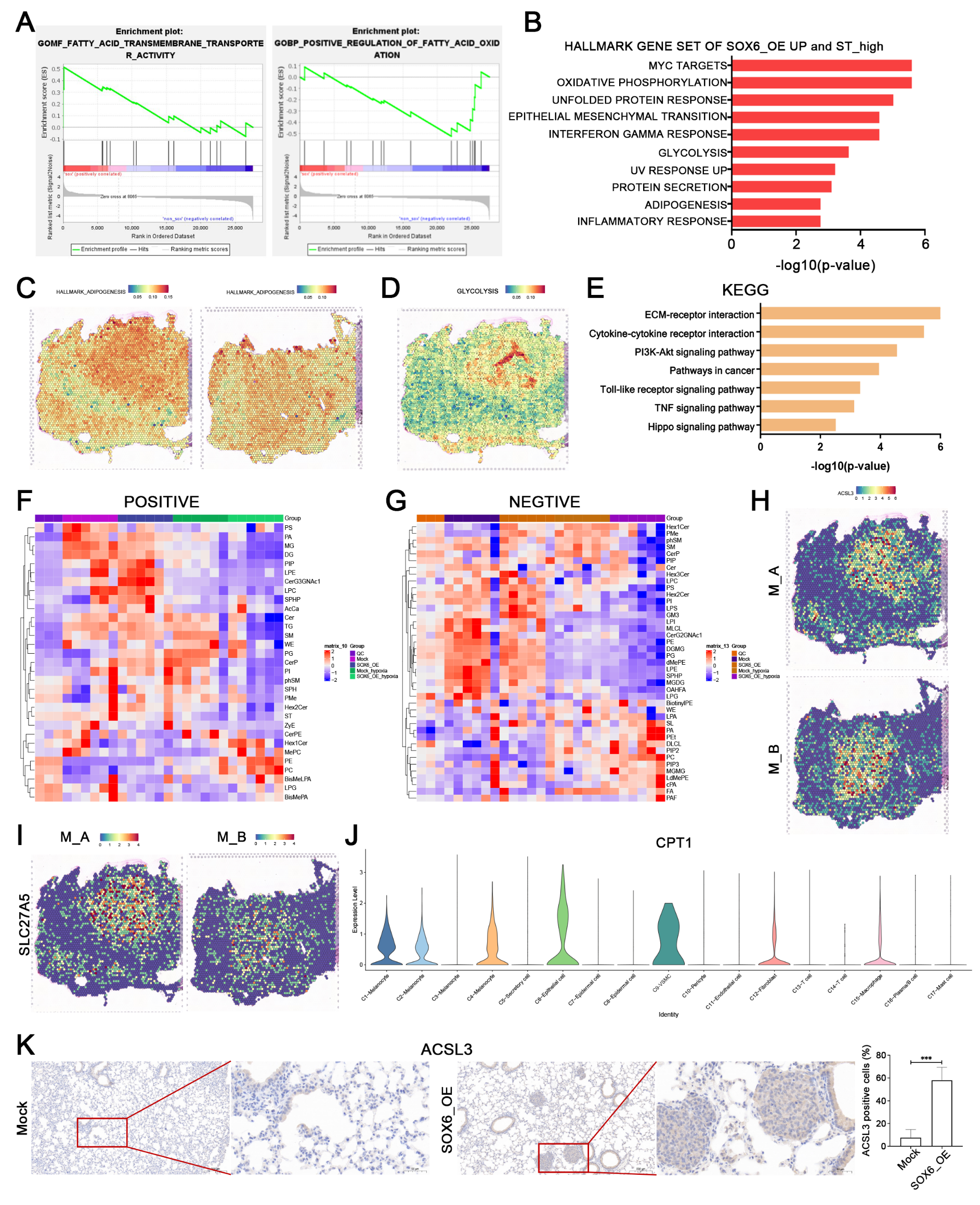


**Supplementary Figure 5. Demonstrating of SOX6_OE in promoting cancer cell invasion.**

1. GSEA analysis of "GOMF_FATTY_ACID_TRANSMEMBRANE_ TRANSPORTER_ACTIVITY" and "GOBP_POSITIVE_RETGULATION_ OF_FATTY_ACID_OXIDATIONL" pathways in the transcriptome of SOX6 overexpressing cells.
2. Hallmark geneset analysis of overlapped genes between spatially highly expressed genes of microsatellite and SOX6 up-regulated genes.

(C) Spatial transcriptomics characterization of significantly up-regulated lipogenesis processes specific to microsatellite lesions in M_A and M_B samples.

(D) Spatial transcriptomics characterization of significantly up-regulated glycolytic processes specific to microsatellite lesions.

(E) KEGG enrichment results of overlapped highly expressed genes from spatial transcriptomics and SOX6 up-regulated genes.

(F-G) Heatmaps display significantly altered lipid species in both positive and negative ionization modes by untargeted fatty acid metabolomic profiling of SOX6_OE cells.

(H-I) High expression of key fatty acid transport genes ACSL3 and SLC27A5 in microsatellite lesions.

(J) Expression of CPT1 in different cell types, particularly up-regulated in C1/C2/C4 cells.

(K) Detection of ACSL3 protein levels in lung metastases of mice injected with SOX6_OE A375 cells via tail vein. Scale bars = 100 μm.
